# Acute Coronary Syndrome Presenting during On- and Off-Hours: Is There a Difference in a Tertiary Cardiovascular Center?

**DOI:** 10.3390/medicina59081420

**Published:** 2023-08-04

**Authors:** Ivan Ilic, Anja Radunovic, Milica Matic, Vasko Zugic, Miljana Ostojic, Milica Stanojlovic, Dejan Kojic, Srdjan Boskovic, Dusan Borzanovic, Stefan Timcic, Dragana Radoicic, Milan Dobric, Milosav Tomovic

**Affiliations:** 1 Institute for Cardiovascular Diseases Dedinje, Heroja Milana Tepića 1, 11000 Belgrade, Serbia; ivan1ilic@yahoo.com (I.I.); milica.matic@ymail.com (M.M.); vaskozugicnk10@gmail.com (V.Z.); kojicdrd@yahoo.com (D.K.); boskovics@hotmail.com (S.B.); d.borzanovic@gmail.com (D.B.); stefan.timcic@icloud.com (S.T.); dragana.kastratovic@yahoo.com (D.R.); drmilandobric@gmail.com (M.D.); milosavtomovic@gmail.com (M.T.); 2 School of Medicine, University of Belgrade, 11000 Belgrade, Serbia; miljanaostojic99@gmail.com (M.O.); sstanojlovicmilica@gmail.com (M.S.)

**Keywords:** acute coronary syndrome, working hours, PCI, STEMI, radial approach

## Abstract

*Background and Objectives*: ACS presents an acute manifestation of coronary artery disease and its treatment is based on timely interventional diagnostics and PCI. It has been known that the treatment and the outcomes are not the same for all the patients with ACS during the working day, depending on the availability of the procedures and staff. The aim of the study was to explore the differences in clinical characteristics and outcomes in patients admitted for ACS during on- and off-hours. *Materials and Methods*: The retrospective study included 1873 consecutive ACS patients admitted to a tertiary, university hospital that underwent coronary angiography and intervention. On-hours were defined from Monday to Friday from 07:30 h to 14:30 h, while the rest was considered off-hours. *Results*: There were more males in the off-hours group (on-hours 475 (56%) vs. off-hours 635 (62%); *p* = 0.011), while previous MI was more frequent in the on-hours group (on 250 (30%) vs. off 148 (14%); *p* < 0.001). NSTEMI was more frequent during on-hours (on 164 (19%) vs. off 55 (5%); *p* < 0.001), while STEMI was more frequent during off-hours (on 585 (69%) vs. off 952 (93%); *p* < 0.001). Patients admitted during on-hours had more multivessel disease (MVD) (on 485 (57%) vs. off 489 (48%); *p* = 0.006), as well as multivessel PCI (on 187 (22%) vs. off 171 (16%); *p* = 0.002), while radial access was preferred in off-hours patients (on 692 (82%) vs. off 883 (86%); *p* = 0.004). Left main PCI was performed with similar frequency in both groups (on 37 (4%) vs. off 35 (3%); *p* = 0.203). Death occurred with similar frequency in both groups (on 17 (2.0%) vs. off 26 (2.54%); *p* = 0.404), while major adverse cardio-cerebral events (MACCEs) were more frequent in the on-hours group (on 105 (12.4%) vs. off 70 (6.8%); *p* = 0.039) probably due to the more frequent repeated PCI (on 49 (5.8%) vs. off 27 (2.6%); *p* = 0.035). *Conclusions*: Patients admitted for ACS during working hours in a tertiary hospital present with more complex CAD, have more demanding interventions, and experience more MACCEs during follow-up mostly due to myocardial infarctions and repeated procedures.

## 1. Introduction

Acute coronary syndrome (ACS) presents an acute manifestation of coronary artery disease, and its treatment is based on timely interventional diagnostics and early mechanical reperfusion of the epicardial coronary artery by percutaneous coronary intervention (PCI) [1]. Every year, about 7 million people in the world are diagnosed with ACS, and acute myocardial infarction (MI) is still one of the leading causes of death in the developed world [2]. 

The spectrum of ACS patients is variable depending on clinical presentation, admission ECG, echocardiography, and the magnitude of myocardial necrosis. While patients with ST elevation myocardial infarction (STEMI) should be treated with primary PCI within 90 min from first medical contact, the timing of the intervention in non-ST elevation MI (NSTEMI) or unstable angina (UA) depends on clinical status, signs of hemodynamic instability, and values of high-sensitivity (hs) troponin [3,4].

Since cardiology is developing rapidly and the number of options to support and treat critically ill ACS patients are growing, there is a concern whether these options are available for every single patient. New techniques require education and experience, and many hospitals cannot provide around-the-clock services in contemporary complex PCI and critical care. Several studies demonstrated conflicting results regarding prognosis, morbidity, and mortality in patients admitted to hospitals during off-hours compared to patients treated during regular working hours [4,5,6,7,8,9]. These discrepancies could be explained by senior staff availability during off-hours, the availability of specific treatments (mechanical circulatory support, intravascular imaging, calcium treatment), the lack of expertise in complex interventional techniques and critical care management, and general staff fatigue during off-hours. 

Therefore, the aim of our study was to explore interventional procedural differences and cardiovascular outcomes in ACS patients admitted and treated during on- and off-hours in tertiary university hospital. 

## 2. Materials and Methods

The study was retrospective, observational, and included 1873 consecutive patients who were admitted for ACS at a tertiary university hospital, Institute for Cardiovascular Diseases (ICVD) Dedinje, Belgrade, Serbia from January 2019 to December 2022. The study included patients that underwent coronary angiography immediately after admission and were further treated with PCI, coronary artery bypass grafting, or just medical therapy. Patients were treated with dual antiplatelet therapy (DAPT) consisting of aspirin and one P2Y12 receptor inhibitor (ticagrelor, prasugrel, or clopidogrel). They were discharged with a recommendation for 12-month DAPT. 

Patient admissions were defined as follows: on-hours—patient’s admission to hospital from 7:30 a.m. to 2:30 p.m. on working days; off-hours—patient’s admission to hospital after 2:30 p.m. until 7:30 a.m. the next morning on working days and 24 h on weekends. On-hours were defined based on the working hours of interventional cardiologists and the cardiology department in our hospital when most of the senior staff is present and supportive services operate with the greatest number of people. During the rest of the day, the cardiology department has an in-house on-duty cardiologist, while the interventional cardiology department has a working team usually made up of junior interventionalists and usually one senior interventional cardiologist, which is far less staff than in the on-hours shift. During weekends, there are on-call teams for interventional cardiology along with in-house cardiologists at the ward, which is quite similar to off-hours during working days. 

Patients with significant valve disease and/or valve surgery were not included in the registry. The study did not include patients that had a significant bleeding episode during hospitalization that required surgical intervention and/or blood transfusion or patients with expected survival due to a noncardiac condition of less than one year. 

Patient data were collected from the hospital database that contained data regarding admissions and office visits for every single patient together with the telephone interview conducted by one of the study’s team members. Patient data were entered into the electronic database created for this study. We collected the following data from the study subjects: Demographic data—previous medical history and risk factors for atherosclerosis. Regarding PCI procedure besides collecting data on CAD distribution, we defined bifurcation lesion PCI (bifurcation with a significant side branch, as perceived by the operator, protected with a wire during PCI and/or a balloon or stent treatment of the side branch). Furthermore, we noted PCI complexity—implantation of more than one stent in the treated vessel, stent diameter of less than three millimeters, use of glycoprotein IIb/IIIa inhibitors, and multivessel PCI—intervention of more than one coronary artery during initial hospitalization. Patients were followed in the hospital and after discharge with Major adverse cardio-cerebral events (MACCEs)—defined as any death, myocardial infarction, cerebrovascular accident (CVA), or repeated PCI during follow-up.

During clinical follow-up after initial admission, patients were contacted by telephone from one of the members of the study team. After obtaining verbal consent to participate in the study, they were interrogated according to the previously defined questionnaire that included information regarding the patient’s vital status, ischemic events—myocardial infarction—CVI and repeated PCI procedures, repeated hospital admissions, and adherence to prescribed antiplatelet medications. The study was conducted according to the declaration of Helsinki and was approved by the institutional review board. 

### Statistical Analysis

Continuous data were presented as means ± standard deviation (SD). Categorical data were presented as numbers (n) or as percentages (%). The T test and Mann–Whitney U test were used for continuous variables, while the chi-squared test and Fisher’s test were used for categorical variables. The effects of patient characteristics on the endpoint of MACCE were explored using multivariate logistic regression, and their impact was expressed as odds ratios with 95% confidence intervals. The forward stepwise regression selection process was used to determine independent predictors of the occurrence of MACCE in a multivariate analysis model. The stepwise selection iteratively selected the most significant variable with multivariate *p*-value < 0.25, to start the model. At each step, another significant variable was added and, after running the model, a check was performed to remove the variable with a multivariate *p*-value > 0.10. This was repeated with the complete set of variables until no more variables could be entered and no variables could be dropped. All *p* values < 0.05 were considered statistically significant. All statistical analyses were performed using IBM SPSS Statistics 19.0 statistical software (IBM Armonk, New York, NY, USA).

## 3. Results

This retrospective, observational study included 1873 consecutive patients who were admitted to ICVD Dedinje during on-hours (*n* = 848) and off-hours (*n* = 1025) in the period of 2019–2022 with a diagnosis of ACS (STEMI, NSTEMI, UA). Initially, 1900 patients were evaluated but follow-up was not available for 27 patients.

The patients admitted during on-hours were less frequently males, had more dyslipidemia, had previous MI more often, but had fewer cerebrovascular accidents (CVA). Both groups had a similar degree of left ventricular dysfunction assessed by echocardiography. Clinical characteristics of patients with ACS admitted during and outside working hours are presented in Table 1.

Since ICVD Dedinje is a referral center and has a 24/7 STEMI service, more STEMI admissions recorded during off-hours would be expected, while NSTEMI and UA were more frequently admitted during on-hours. Regarding door-to-balloon time (D2B), it was only recorded in STEMI patients and they were similar in both groups (on-hours 47 ± 33 min vs. off-hours 44 ± 29 min, *p* = 0.604). STEMI patients had higher values of hsTroponin, but the patients in both groups had a similar duration of hospital stay and incidence of in-hospital death. Also, there was no difference between the study groups regarding the need for surgical revascularization, as can be seen in Table 2.

Multivessel coronary disease was recorded more often in patients admitted during working hours. The radial approach was used more often in patients admitted during off-hours. Multivessel PCI was performed more often during regular working hours, but there were no differences between the groups regarding PCI complexity (left main intervention, bifurcation, long lesions, small vessels, saphenous vein graft interventions). Characteristics of coronary disease and interventional procedures are presented in Table 3.

The mean follow-up was 30 months ± 7 months. During follow-up, 1216 patients were monitored. Adverse cardiovascular events defined as MACCE—any death, MI, CVA, repeated PCI, or coronary bypass grafting (CABG) were more frequent in the on-hours group mostly due to the notable difference in the rate of repeated PCI in patients admitted during working hours. Major adverse cardiovascular events are shown in Table 4. 

**Figure 1:**
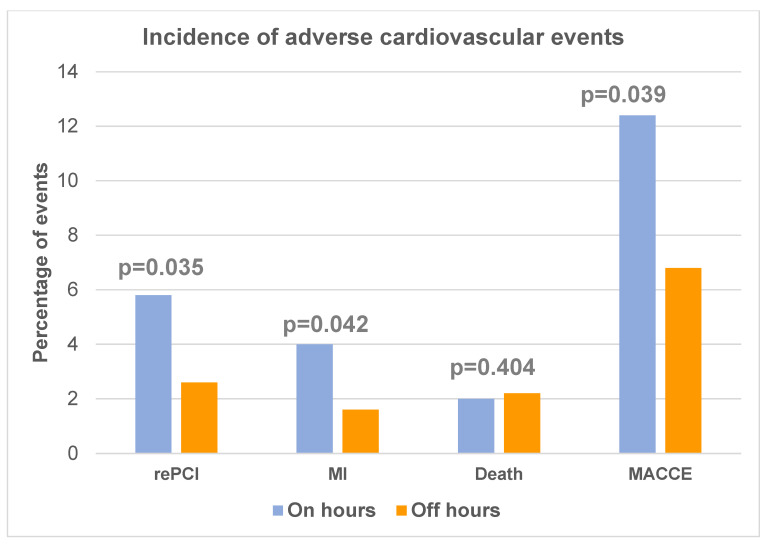
Incidence of adverse cardiovascular events in patients’ groups.

Multivariable logistic regression analysis revealed that age and multivessel disease remained an independent predictor of MACCE in our patient group. Admission during working hours only had a borderline association in univariate analysis, which was lost in a multivariable model. The results of multivariable logistic regression analyses to identify predictors of MACCE are presented in Table 5 and Figure 1.

## 4. Discussion

We presented an analysis of patients admitted for ACS to a tertiary university hospital that were divided according to the time of admission into on- and off-hours groups. We found that the patients that were admitted during on-hours were more frequently presenting with ACS forms other than STEMI, more often had previous myocardial infarction, had more MVD, and underwent multivessel PCI. These patients during follow-up had more MACCEs mostly due to the higher rate of repeated PCI. Despite the higher incidence of MACCE in this group, in multivariable analysis, only MVD was found to be an independent predictor of MACCE, while the time of admission only had a borderline association with MACCEs in a univariate model. 

The working hours and off-hours were defined according to the working hours of the interventional cardiology department when most of the senior operators were present. This definition is based on the reduced exposure time to radiation according to local laws, which define the maximum time to be spent in the area where radiation is used. The off-hours during the week and weekends have the same reduced staff availability, which caused us to put them together in the off-hours group. This may be different in other studies because of different local laws and hospital protocols like in a study by Marume et al., where on-hours were defined from 8:30 AM to 05:15 PM from Monday to Friday, and a study by Enezate et al., where on-hours were defined from 08:00 AM till 05:00 PM, while in both studies, off-hours included the rest of the workdays and weekends [10,11]. However, our definition had similar characteristics regarding the availability of senior staff and complex procedures and supportive services. 

There were significant differences between on- and off-hours-admitted patients regarding gender (male 56% (475) vs. 62% (635), *p* = 0.011), presence of dyslipidemia (46% (390) vs. 34% (348), *p* ˂ 0.001), previous history of MI (30% (250) vs. 14% (148), *p* ˂ 0.001), presence of CVA (5% (41) vs. 7% (75), *p* = 0.033), and clinical presentation of ACS (UA 12% (99) vs. 2% (18), *p* ˂ 0.001, STEMI 69% (585) vs. 93% (952), *p* ˂ 0.001, NSTEMI 19% (164) vs. 5% (55), *p* ˂ 0.001). More female patients in the on-hours group might cause a higher rate of MACCEs. The large registries showed confounding results regarding sex differences in MACE rates after ACE. A large Australian registry that followed more than 9000 ACS patients demonstrated higher MACE rates among women at six months after the first event [12]. A large Indian registry showed that females less likely present with STEMI and have a higher incidence of short-term MACE, but after adjustments, these differences disappeared, similar to EXAMINE trials where sex differences did not have an influence on cardiovascular events [13,14]. The differences in initial presentation could play a pivotal role in causing differences in MACCE incidence. A large registry from Canada showed that mortality rates were higher in patients with NSTEMI compared to UA and STEMI [14,15]. The greater number of non-STEMI patients during on-hours could be caused by the fact that ICVD serves as a referral center for patients with CAD and that most of the transfers would be patients that were admitted for the non-STEMI form of ACS and usually transferred during on-hours the next day. Also, we serve as a 24/7 STEMI hospital, so admissions during off-hours are more frequently patients with STEMI. 

Previous studies demonstrated that the short- and long-time mortality rates of patients admitted off-hours (weekends) were often higher [16,17]. Based on the results of single-centered studies and a large meta-analysis, increased mortality can be attributed to the longer PCI-related delay that is more frequent during off-hours [18,19,20,21]. Furthermore, the delay can be due to several factors more frequently encountered during off-hours like the decreased number of physicians and nursing staff, the lack of expertise in complex clinical scenarios, the decreased availability of complex techniques like intravascular imaging or mechanical circulatory support, and factors such as sleep deprivation and fatigue [22,23]. A recent study found that patients with acute MI in regions with a lower number of cardiologists per number of inhabitants had a higher 30-day mortality, suggesting that the availability of cardiologists in the regional system of care may affect the outcomes of patients with acute MI [24].

Regarding the D2B time in our registry, there was no difference between on- and off- hours. This may be due to the hospital’s organization for treating STEMI patients. There is an entire interventional team consisting of two interventional cardiologists and nurse/technicians on-duty in the hospital when 24 h on-call for the city’s STEMI network. This way, the hospital can treat two STEMI patients at the same time. Previously, there were interventional teams in an on-call mode, but due to the transportation issues, we noticed that our D2B times were worse than expected and discrepant with the current guidelines [3]. From 2018, the hospital management decided to have interventional teams in the hospital during the days of duty for the STEMI network when most of our STEMI patients were admitted. 

Previous multicenter randomized trials [25,26,27] have undoubtedly shown that transradial artery access for percutaneous coronary intervention is associated with lower bleeding and vascular complications than transfemoral artery access, especially in patients with acute coronary syndromes. Radial access dominated in both groups, but it was more often used in the off-hours group ((82%) 692 vs. (86%) 883, *p* = 0.004). This could be related to the increased incidence of STEMI patients in the off-hours group [19] but can also be due to the increased complexity of interventions (multivessel PCI, more stents implanted) requiring a larger guiding catheter size obtained by transfemoral access. On the other hand, objective indicators of PCI complexity like LM PCI and bifurcation PCI did not occur more frequently in the on-hours group.

As we previously mentioned, a key difference observed between the on-hours and off-hours groups presenting with ACS was that there were more patients with NSTEMI and unstable angina in the on-hours group. Consequently, we can expect that the patients that were admitted during on-hours had more MVD (57% (485) vs. 48% (489), *p* = 0.006) and underwent multivessel PCI (22% (187) vs. 16% (171) *p* = 0.002). Also, we noticed borderline significant differences in the number of procedures with more than one stent (41% (344) vs. 37% (352)*, p* = 0.089). These factors can account for the greater incidence of repeated revascularization during follow-up, contributing critically to the higher incidence of MACCE in this [28,29,30,31].

Multivariable analysis demonstrated that on- vs. off-hours admission and treatment were not independently associated with cardiovascular adverse events. On the other hand, patients’ age and MVD remained an independent predictor of MACCE. This finding is concordant with the findings from previous studies of ACS population. Age has been traditionally associated with adverse outcomes in ACS. Data from the GRACE registry have identified age as a determining factor of adverse events during follow-up [31]. Parodi et al. in a registry of ACS patients undergoing PCI found that age and MVD were identified as independent predictors of adverse events [32]. On the other hand, the association of MVD and MACCE events can be explained by powerful effects of advanced CAD on outcomes regardless of the kind of treatment administered [33,34].

Some limitations of our study should be noted. The study was retrospective, and the outcomes were recorded based on patients’ accounts and available medical data. The events were not adjudicated by an independent observer. The specific organization of the institution with interventional teams in the hospital during STEMI duties may obscure the difference between on- and off-hours regarding patients’ outcomes and adverse events.

## 5. Conclusions

Despite advances in the management of ACS, there continues to be a significant risk of recurrent cardiovascular events following initial presentation. In our single-centered study, the patients admitted for ACS during on-hours typically presented with more complex CAD, ultimately leading to more demanding interventions, and a higher adverse cardiovascular events rate attributed to more myocardial infarctions and repeated interventions during follow-up.

## Figures and Tables

**Table 1:** Clinical characteristics of patients with ACS admitted during and outside working hours.

Variable	ON *n* = 848	OFF *n* = 1025	*p* Value
Age (years)	63 ± 11	61 ± 10	0.241
Males n (%)	475 (56)	635 (62)	0.011
Heredity n (%)	296 (35)	379 (37)	0.266
Hypertension n (%)	644 (76)	738 (72)	0.064
Diabetes mellitus n (%)	203 (24)	215 (21)	0.087
Dyslipidemia n (%)	390 (46)	348 (34)	<0.001
PAD n (%)	43 (5)	45 (4)	0.255
Smoking n (%)	356 (42)	474 (46)	0.167
Previous MI n (%)	250 (30)	148 (14)	<0.001
Previous CVA n (%)	41 (5)	75 (7)	0.033
Previous PCI n (%)	128 (15)	128 (12)	0.124
Previous CABG n (%)	40 (4)	38 (4)	0.451
BMI (kg/m^2^)	29.3 ± 6.9	28.5 ± 7.4	0.554
LVEF (%)	38.6 ± 11.3	37.8 ± 15.1	0.568

BMI—body mass index; CABG—coronary artery bypass grafting; CVA—cerebrovascular accident; LVEF—left ventricular ejection fraction; MI—myocardial infarction; PAD—peripheral arterial disease.

**Table 2:** Clinical presentation and treatment of patients with ACS admitted during on-hours vs. off-hours.

Variable	ON *n* = 848	OFF *n* = 1025	*p* Value
Unstable angina (%)	99 (12)	18 (2)	<0.001
STEMI n (%)	585 (69)	952 (93)	<0.001
NSTEMI n (%)	164 (19)	55 (5)	<0.001
CABG during hosp. n (%)	7 (0.8)	5 (0.5)	0.263
No treatment n (%)	127 (15)	138 (13)	0.187
Days in hospital (days)	3.0 ± 2.6	3.6 ± 4.0	0.056
Maximum hs troponin (ng)	41,789 ± 84,560	58,356 ± 62,079	0.050
Death during hosp. n (%)	5 (0.6)	14 (1.4)	0.071

CABG—coronary artery bypass grafting; NSTEMI—non-ST elevation myocardial infarction; PCI—percutaneous coronary intervention; STEMI—ST elevation myocardial infarction.

**Table 3:** Characteristics of coronary artery disease and intervention in patients with ACS admitted during on-hours vs. off-hours.

Variable	ON *n* = 848	OFF *n* = 1025	*p* Value
No significant disease n (%)	174 (20)	96 (9)	<0.001
Single vessel disease n (%)	363 (43)	536 (52)	<0.001
MVD n (%)	485(57)	489(48)	0.006
* Vessels treated			
LAD n (%)	327 (38)	475(46)	0.001
Cx n (%)	228 (27)	297 (29)	0.256
RCA n (%)	266 (31)	538 (52)	<0.001
SVG n (%)	21 (2)	19 (2)	0.506
Radial access n (%)	692 (82)	883 (86)	0.004
Multivessel PCI n (%)	187 (22)	171 (16)	0.002
LM PCI n (%) (ng)	37 (4)	35 (3)	0.203
Bifurcation PCI n (%)	110 (13)	124 (12)	0.409
More than one stent n (%)	341 (41)	352 (37)	0.089
Stent diameter < 3.0 mm n (%)	249 (29)	276 (27)	0.331

* Vessels treated during initial hospitalization. Cx—circumflex; LAD—left anterior descending; LM—left main; PCI—percutaneous coronary intervention; RCA—right coronary artery; SVG—saphenous vein graft.

**Table 4:** Major adverse cardiovascular events during follow-up in patients with ACS admitted during on-hours vs. off-hours.

Variable	ON *n* = 848	OFF *n* = 1025	*p* Value
Death n (%)	17 (2.0)	26 (2.54)	0.404
Myocardial infarction n (%)	34 (4.0)	17 (1.6)	0.042
CVA n (%)	0 (0)	0 (0)	--------
Repeated PCI n (%)	49 (5.8)	27 (2.6)	0.035
CABG n (%)	5 (0.6)	3 (0.4)	0.524
MACCE n (%)	105 (12.4)	70 (6.8)	0.039

CABG—coronary artery bypass grafting; CVA—cerebrovascular accident; MACCE—major adverse cardio-cerebral events.

**Table 5:** Univariate and multivariable logistic regression analyses to identify predictors of MACCE.

	Univariate		Multivariate	
	HR [95% CI]	*p*-Value	HR [95% CI]	*p*-Value
Age	**1.025 [1.004–1.048]**	**0.021**	**1.024 [1.283–3.296]**	**0.035**
Male sex	1.310 [0.820–2.090]	0.258	- - -	- - -
Diabetes	1.375 [0.792–2.386]	0.258	- - -	- - -
LVEF	0.994 [0.971–1.018]	0.626	- - -	- - -
STEMI	0.729 [0.405–1.312]	0.292	- - -	- - -
Multivessel disease	**2.141 [1.339–3.423]**	0.001	**2.056 [1.283–3.296]**	**0.003**
On hours admission	1.755 [0.994–3.099]	0.052	1.375 [0.992–2.386]	0.258

CI—confidence interval; HR—hazard ratio; LVEF—left ventricular ejection fraction; STEMI—ST elevation myocardial infarction.

## Data Availability

Data available on request due to restrictions eg privacy or ethical.
